# Anacardic acid attenuates pressure‐overload cardiac hypertrophy through inhibiting histone acetylases

**DOI:** 10.1111/jcmm.14181

**Published:** 2019-02-03

**Authors:** Shuo Li, Bohui Peng, Xiaomei Luo, Huichao Sun, Chang Peng

**Affiliations:** ^1^ Department of Pediatrics Affiliated Hospital of Zunyi Medical University ZunYi Guizhou China; ^2^ Department of Physiology Zunyi Medical University Zunyi Guizhou China; ^3^ Heart Center Children's Hospital of Chongqing Medical University Chongqing China

**Keywords:** anacardic acid, cardiac hypertrophy, histone acetylation, thoracic aorta constriction

## Abstract

Cardiac hypertrophy has become a major cardiovascular problem wordwide and is considered the early stage of heart failure. Treatment and prevention strategies are needed due to the suboptimal efficacy of current treatment methods. Recently, many studies have demonstrated the important role of histone acetylation in myocardium remodelling along with cardiac hypertrophy. A Chinese herbal extract containing anacardic acid (AA) is known to possess strong histone acetylation inhibitory effects. In previous studies, we demonstrated that AA could reverse alcohol‐induced cardiac hypertrophy in an animal model at the foetal stage. Here, we investigated whether AA could attenuate cardiac hypertrophy through the modulation of histone acetylation and explored its potential mechanisms in the hearts of transverse aortic constriction (TAC) mice. This study showed that AA attenuated hyperacetylation of acetylated lysine 9 on histone H3 (H3K9ac) by inhibiting the expression of p300 and p300/CBP‐associated factor (PCAF) in TAC mice. Moreover, AA normalized the transcriptional activity of the heart nuclear transcription factor *MEF2A*. The high expression of cardiac hypertrophy‐linked genes (*ANP, β‐MHC*) was reversed through AA treatment in the hearts of TAC mice. Additionally, we found that AA improved cardiac function and survival rate in TAC mice. The current results further highlight the mechanism by which histone acetylation is controlled by AA treatment, which may help prevent and treat hypertrophic cardiomyopathy.

## 1 INTRODUCTION

Recently many studies have revealed the crucial role of chromatin remodelling particularly histone acetylation in cells during the regulation of gene expression in cardiovascular tissues. Histone acetylase (HAT) helps translocate acetyl groups from acetyl coenzyme A onto the ϵ‐amino group of lysine residues within nucleosomal histone tails, which neutralizes the charges carried by amino acids. This altered charge of the histone tail promotes chromatin relaxation and thus creates a local environment that accommodates the transcriptional machinery.^1^ To date, epigenetic gene regulation has been shown to actively participate in the pathogenesis of cardiovascular diseases.^2^, ^3^ Cardiac hypertrophy is an important pathological process and is considered the early stage of heart failure. Recently, HAT and histone deacetylase (HDAC) were shown to be involved in the modulation and regulation of hypertrophic responses in heart dysfunction.^4^, ^5^, ^6^ We previously demonstrated that the imbalance of histone acetylation modification induced by HAT is involved in the development and progression of cardiac hypertrophy and that anacardic acid (AA) can decrease alcohol‐mediated cardiac hypertrophy.^7^ Unfortunately, the mechanism of AA underlying the Chinese herb extract‐mediated attenuation of cardiac hypertrophy caused by transverse aortic constriction (TAC) remains unclear. Here, we aimed to assess the potential of AA as a pan‐HAT inhibitor that corrects cardiac hypertrophy by suppressing HAT in an overload‐mediated cardiac hypertrophy model. These studies were carried out to identify strategies to decrease pressure overload‐induced cardiac hypertrophy.

## 2 METHODOLOGY

### 2.1 Experimental mice

Sterile‐ or pathogen‐free 10‐ to 12‐week‐old Kunming mice (both male and female) with a body mass of 25‐30 g were purchased from the Experimental Animal Center at Zunyi Medical University (Guizhou, China). The animal trials used in the experiments were approved by the Animal Care and Use Committee of Zunyi Medical University. Mice were kept under fully controlled conditions (22 ± 1°C, 55% ± 5% humidity) and allowed food ad libitum along with equal light:dark cycles (12 h:12 h). Mice were exposed to pressure overload through thoracic aortic banding (TAB) (≈70% thoracic aortic diameter). TAC mice were given AA which is a pan‐HAT inhibitor. Dimethylsulphoxide was used to dissolve AA for further preparations of 1 mg/ml and finally stored at 4°C. AA (3.75 ml/kg) was intraperitoneally injected into TAC mice every third day for up to 3 weeks after operation. Killing of mice was carried out by CO_2_ narcosis, and finally, the heart tissues were collected for further analyses.

### 2.2 Detection of HAT activity

The mouse myocardial tissues were homogenized to extract nucleoproteins using a Nuclear Extract Kit (Invent, Minnesota, USA) following the manufacturer's instructions. The HAT activity of the protein extracts was examined colourimetrically using a HAT assay kit (GenMed, Shanghai, China).

### 2.3 Chromatin immunoprecipitation (ChIP)

The mouse heart tissue homogenates were placed in 1% formaldehyde for crosslinking of DNA‐protein complexes. After crosslinking, the DNA was sheared through sonication followed by DNA‐protein complex precipitation using monoclonal antibodies (anti‐MEF2A, anti‐p300, anti‐PCAF and anti‐GCN5) (ChIP grade, Abcam, Cambridge, England). A DNA purification kit (Merck Millipore, Darmstadt, Germany) was used to extract the pure DNA molecules. All experiments had both positive control (precipitated by anti‐RNA polymerase II antibody) and negative control (precipitated by normal mouse IgG) groups. Quantitative real‐time PCR was performed after ChIP assays were conducted using a ChIP assay Kit (Merck Millipore, Darmstadt, Germany).

### 2.4 Immunoblotting

The mouse myocardial tissues were harvested to extract the nucleoproteins mentioned above and separated on 8/12% sodium dodecyl sulphate polyacrylamide gels through electrophoresis and blotted onto polyvinylidene difluoride (PVDF) membranes (Merck Millipore, Darmstadt, Germany). These PVDF blots were probed against rabbit polyclonal antibodies [acetylated lysine 9 on histone H3 (H3K9ac), atrial natriuretic peptide (ANP), foetal isoform of myosin heavy chain (β‐MHC), α‐actin, p300 and PCAF] (Abcam, Cambridge, UK, 1:1,000 dilution) or rabbit polyclonal antibody against histone H3 and β‐actin (Beyotime, Shanghai, China, 1:1,000 dilution) in Tris‐buffered saline with Tween 20 (TBST) plus 5% non‐fat milk at 4°C overnight. HRP‐conjugated goat anti‐rabbit antibody (Santa Cruz Biotechnology, Texas, USA) was used as the secondary antibody. After the PVDF membranes were scanned, the bands were subjected to analysis using the Quantity One (Version 4.4) software package (Bio‐Rad, CA, USA).

### 2.5 Haematoxylin and Eosin (H&E) staining

Mouse heart tissues were collected and placed in 4% paraformaldehyde for 24 hours and were later transferred to 70% ethyl alcohol. Each heart tissue was placed into processing cassettes, passed through a gradient alcoholic series for dehydration and finally embedded in paraffin wax. Prior to immunostaining, heart tissue sections (5 μm) were dewaxed using xylene, rehydrated through reverse alcoholic gradient washed with PBS, and finally stained using haematoxylin and eosin.

### 2.6 Echocardiography

Trans‐thoracic echocardiograms of conscious‐sedated mice were performed following the previously described methods^8^, ^9^.

### 2.7 Statistical analysis

The data obtained in this study were subjected to statistical analyses and are shown as the mean ± standard deviation (SD). One‐way analysis of variance (1‐way ANOVA) and LSD‐*t* tests were applied to determine the significance of the results, where *P* < 0.05 was defined as statistically significant.

## 3 RESULTS

### 3.1 Pressure overload‐induced cardiac hypertrophy caused by TAB in mice

**Figure 1:**
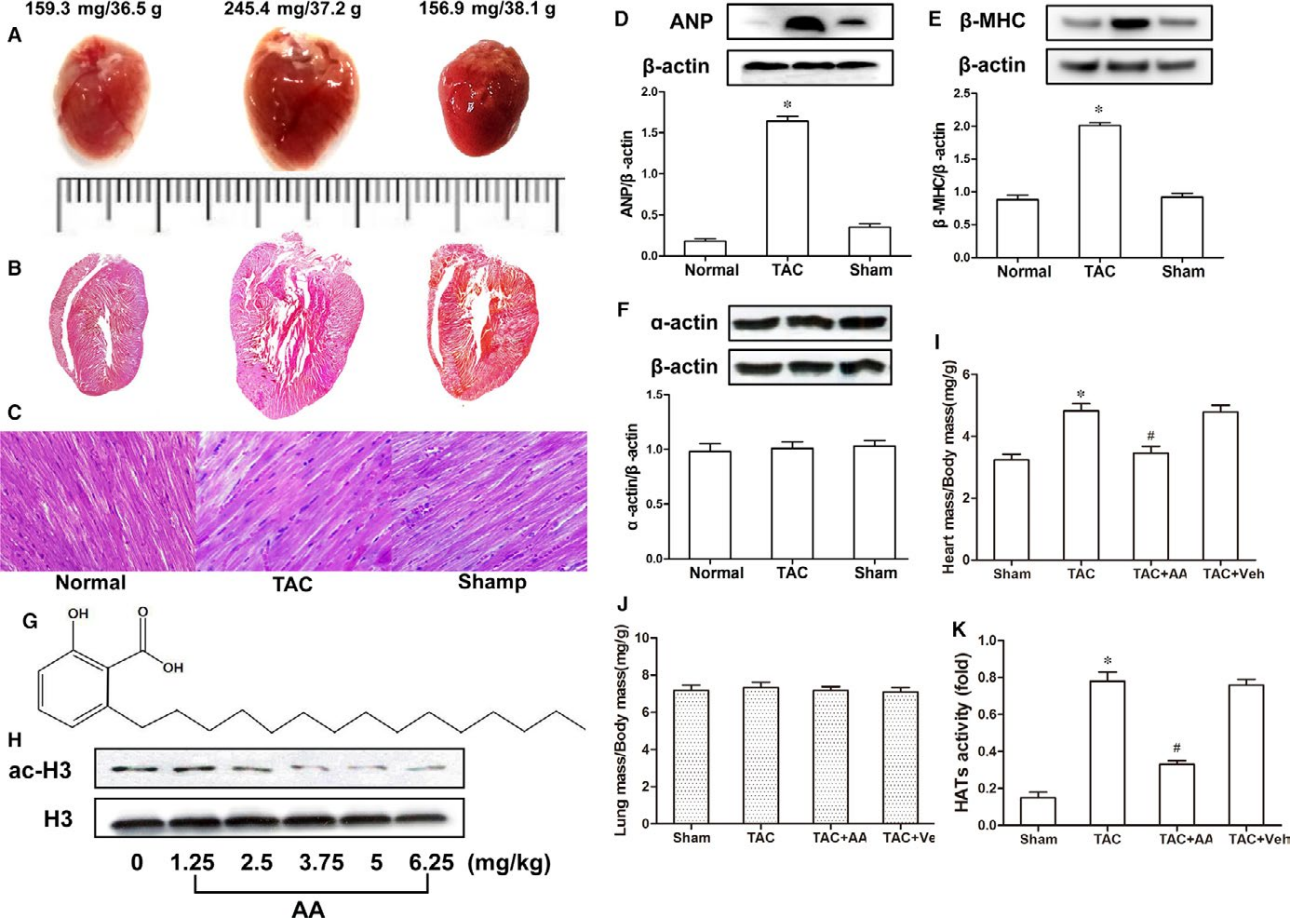
Anacardic acid (AA) blunts pressure overload‐induced hypertrophy in mouse hearts. Stereoscope and haematoxylin and eosin staining analyses of the mouse hearts treated by thoracic aortic banding (TAB) showed that they were significantly inflated compared with those of the sham group, but myocardial hypertrophy, particularly in the left ventricular and interventricular septum, was observed in the transverse aortic constriction (TAC) mice. (A) Characteristic images of complete mouse hearts. (B) Haematoxylin and eosin‐stained longitudinal sections. (C) Longitudinal view of cardiomyocytes. Scale = 20 μm in C. Additionally, the myocardial hypertrophy biomarkers atrial natriuretic peptide (ANP) and myosin heavy chain (β‐MHC) were increased significantly in the TAC mouse hearts compared with those of the sham group (D and E), but the expression levels of α‐actin remained the same in similar samples (F). (G) The chemical structure of the histone acetylase (HAT) inhibitor AA. (H) Different concentrations of AA were used to determine the optimal exposure dose of AA, and 3.75 mg/kg was selected based on the ac‐H3 level. (I‐J) Quantitative analysis of cardiac mass index and lung mass index. (K) The HAT activity was increased significantly in the TAC mouse hearts compared to that of the sham group, but HAT activity was reduced in the hearts of mice treated with AA. *P < 0.05 vs the sham group, ^#^ *P* < 0.05 vs TAC (n = 6)

To investigate the TAC‐induced myocardial hypertrophy in mouse hearts, we exposed the mice to pressure overload through TAB, and a histological examination was performed on the mouse heart tissues. The TAC mouse heart data obtained from stereoscopic analysis and haematoxylin and eosin staining showed apparent enlargement compared with those of the sham group. Myocardial hypertrophy was present, especially in the interventricular septum and left ventricle (Figure 1A, 1). Moreover, haematoxylin and eosin staining revealed diffuse hypertrophy by myocytes, while nuclei in the myocardial cells were significantly inflated compared with those of the sham group (Figure 1C). To assess the body‐weight effect on heart size, we examined the cardiac mass index (CMI) and lung mass index (LMI) in this study. The results demonstrated that CMI was apparently increased in TAC mice compared to sham group mice, while LMI had no defined difference in the same mouse hearts (Table 1). To further explore the changes in myocardial hypertrophy at the molecular and gene levels, we assessed biomarkers of myocardial hypertrophy in the TAC mouse hearts. The data showed that during hypertrophic growth in the hearts, expression of the β‐MHC gene was significantly enhanced, while ANP was improved (Figure 1D, 1). However, the expression of α‐actin was unchanged in the same heart tissues (Figure 1F).

**Table 1:** The comparison of CMI and LMI in the hearts of mice (n = 6)

	HW (g)	LW (g)	BW (g)	HW/BW (mg/g)	LW/BW (mg/g)
Normal	0.13 ± 0.01	0.28 ± 0.04	38.35 ± 3.64	3.39 ± 0.21	7.16 ± 0.62
TAC	0.18 ± 0.02	0.30 ± 0.04	39.38 ± 5.16	4.71 ± 0.52*	7.77 ± 0.89
Sham	0.15 ± 0.02	0.29 ± 0.06	42.22 ± 6.15	3.59 ± 0.26	6.82 ± 0.94
*P* value	－	－	－	0.01	0.20

CMI, cardiac mass index, LMI, lung mass index, TAC, thoraic aorta coarctation.

* *P* < 0.05 TAC vs normal group.

### 3.2 AA inhibits pressure overload‐induced cardiac hypertrophy

To examine the effect of the HAT inhibitor AA on pressure overload‐mediated cardiac hypertrophy, we exposed the mice to TAB, and the molecular structural formula of Chinese AA herbal extracts was defined (Figure 1G). The optimal exposure dose of AA was first determined. TAC mice were injected intraperitoneally with various concentrations of AA (0, 1.25, 2.5, 3.75, 5.0 and 6.25 mg/kg) based on previous reports.^10^ An optimum concentration (3.75 mg/kg) was defined based on the ac‐H3 level in the hearts of TAC mice (Figure 1H). After operation, mice were randomly injected intraperitoneally with either AA or vehicle (Veh). Another group of mice was assigned as the sham operation group, which was injected with Veh every third day for up to 3 weeks. We observed pressure overload‐induced cardiac hypertrophy mediated by TAB in mouse hearts, and administration of AA (3.75 mg/kg) significantly suppressed hypertrophic growth measured as the heart mass normalized to the body mass (Figure 1I), but the lung mass showed no change in the same samples (Figure 1J).

### 3.3 The HAT activity and echocardiography results in the hypertrophic hearts induced by TAC

**Figure 2:**
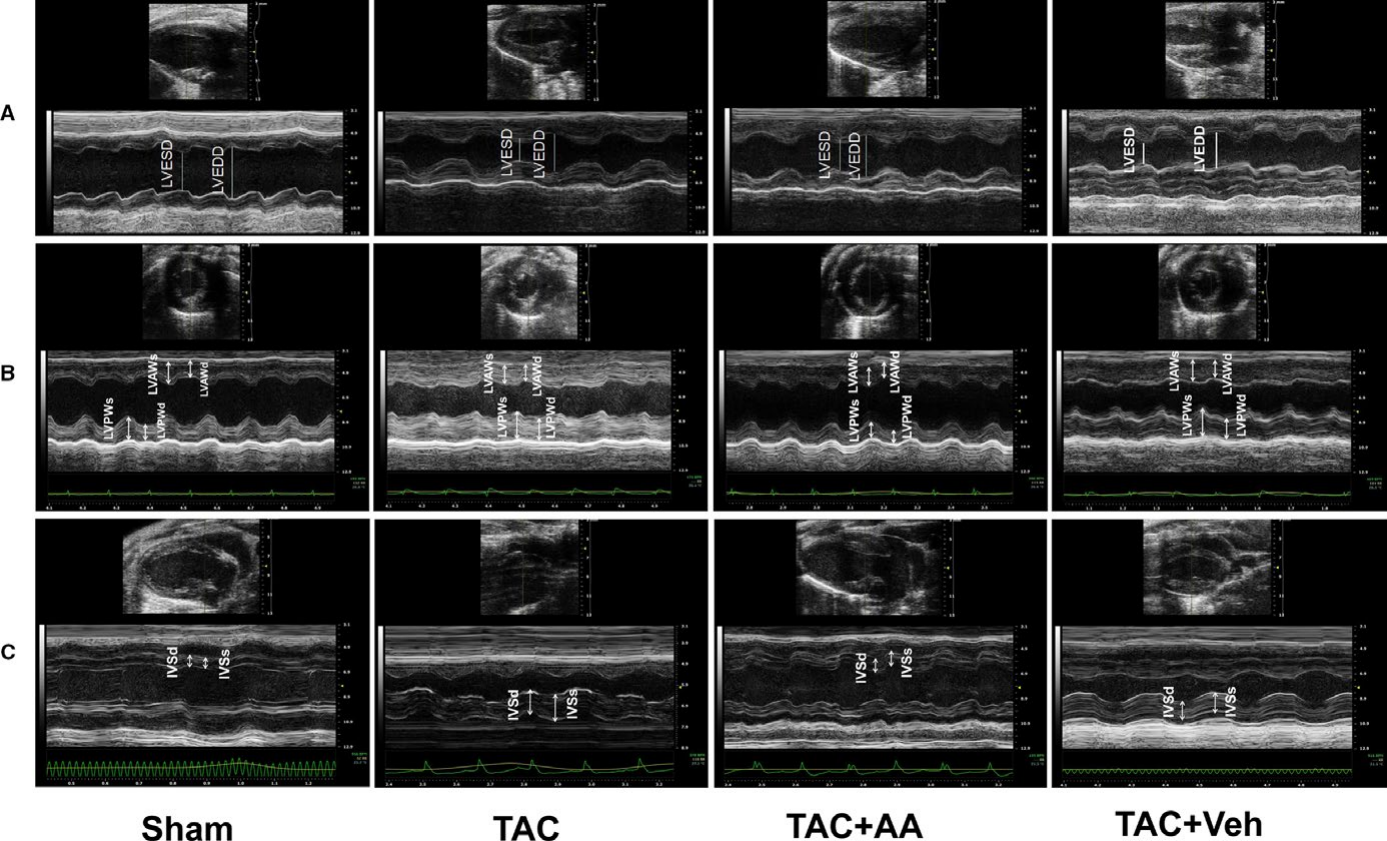
Echocardiographic images of transverse aortic constriction (TAC) mice. Characteristic M‐mode echocardiograms from TAC mice with or without anacardic acid treatment (n = 6). (A) Left ventricular end diastolic dimension (LVEDD) and left ventricular end systolic dimension (LVESD) images of the left ventricular long axis view, (B) LVAW and LVPW images of the left ventricular short axis view and (C) interventricular septum (IVS) images of the left ventricular long axis view. LVEDD: left ventricular end diastolic dimension, LVESD: left ventricular end systolic dimension, LVAW: left ventricular posterior wall, LVPW: left ventricular posterior wall, IVS: interventricular septum

Some evidence has suggested that the imbalance of histone acetylation modification is involved in cardiac hypertrophy induced by TAC. To determine whether hyperacetylation of H3K9ac induced by HAT might be a vital factor in promoting myocardial hypertrophy, we tested the HAT activity in hypertrophic mouse hearts. Colorimetric assays revealed that HAT activity was significantly increased in TAC mice hearts (Figure 1K). Meanwhile, the echocardiography results also demonstrated that AA could attenuate pressure overload cardiac hypertrophy induced by TAB in the mouse hearts (Figure 2A‐C). These data suggested that AA attenuated pressure overload‐induced cardiac hypertrophy. However, the potential mechanisms remain unknown. Next, we explored the potential mechanisms from an epigenetic perspective.

### 3.4 The regulatory relationship between HAT and the transcription factor *MEF2A*

**Figure 3:**
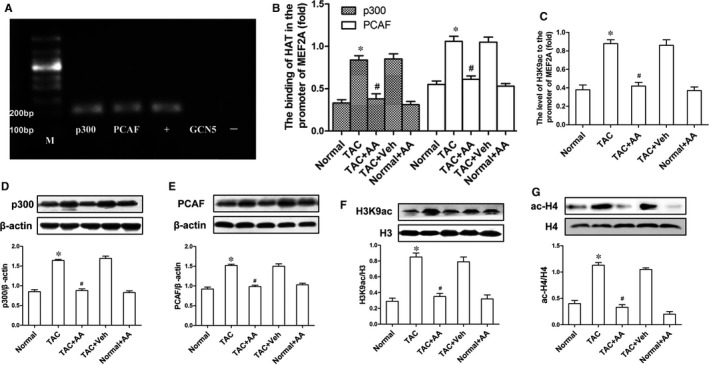
Anacardic acid (AA) attenuates hyperacetylation of H3K9ac and inhibits the overexpression of histone acetylase (HAT) induced by transverse aortic constriction (TAC). ChIP‐PCR results demonstrated that p300 and PCAF, but not general control nonderepressible‐5 (GCN5)*,* could bind to the *MEF2A *promoter, (+) positive control, amplified the DNA fragment precipitated by anti‐RNA polymerase II antibody; (−) negative control, amplified the DNA fragments precipitated by normal mouse IgG. (B) The binding of p300 and PCAF at the promoter region of *MEF2A* showed a significant increase in TAC mice, while this binding was reduced in the TAC mice treated with AA. (C) The level of H3K9ac at the *MEF2A* promoter was the same as that in (B). (D and E) Western blots showed that AA normalized the overexpression of both p300‐HAT and PCAF‐HAT in the TAC mice. (F and G) Immunoblots clearly showed a sharp decrease in hyperacetylation of H3K9ac and ac‐H4 induced by TAC in the hearts of mice treated with AA. *P < 0.05 vs the normal group. ^#^ *P* < 0.05 vs the TAC group (n = 6)

We further explored the effects of p300‐HAT and PCAF‐HAT, which control histone acetylation by regulating stress responses through *MEF2A* transcriptional activity in the mouse heart. The regulatory relationship between HAT and *MEF2A *was tested by ChIP‐PCR, and p300‐HAT and PCAF‐HAT efficiently bound the *MEF2A *promoter; however, GCN5‐HAT could not bind to the *MEF2A *promoter (Figure 3A), indicating that p300‐HAT and PCAF‐HAT were involved in modulating *MEF2A* transcription.

### 3.5 AA reverses hyperacetylation of H3K9ac by inhibiting p300 and PCAF and normalizing the transcriptional activity of *MEF2A*

To investigate the mechanisms by which AA decreases cardiac hypertrophy in TAC mice, we performed ChIP‐Q‐PCR experiments. The ChIP‐Q‐PCR assays showed that AA significantly reduced the binding of p300‐HAT and PCAF‐HAT at the *MEF2A *promoter in TAC + AA mice compared with TAC + Veh mice (Figure 3B). Therefore, we have suggested that the H3K9ac level on the *MEF2A *promoter also decreased in TAC + AA mice compared to TAC + Veh mice. As expected, similar results were obtained when we examined H3K9ac level on the *MEF2A *promoter in the same samples (Figure 3C). Meanwhile, the Western blot data showed that decreases in p300 and PCAF were observed in the TAC mice treated with AA compared to the TAC mice, but there was no significant change in the TAC + Veh mice (Figure 3D,E). In previous experiments, we demonstrated that p300 and PCAF are involved in controlling the transcription of *MEF2A* using ChIP‐PCR and that AA could inhibit the overexpression of p300‐HAT and PCAF‐HAT in the mouse heart. Thus, we have suggested that the level of histone acetylation was inhibited in the same samples. Western blots demonstrated that AA attenuated the hyperacetylation of H3K9ac at the translational level in TAC mice, as expected (Figure 3F). In addition, ac‐H4 was tested in the mouse heart, and immunoblot data showed that the ac‐H4 level was increased in the hearts of mice treated with TAB compared to that of the sham group. Interestingly, AA significantly decreased the ac‐H4 level in the hearts of TAC mice (Figure 3G).

### 3.6 AA decreases the transcriptional activity of *MEF2A* and normalizes the overexpression of downstream cardiac hypertrophic genes

**Figure 4:**
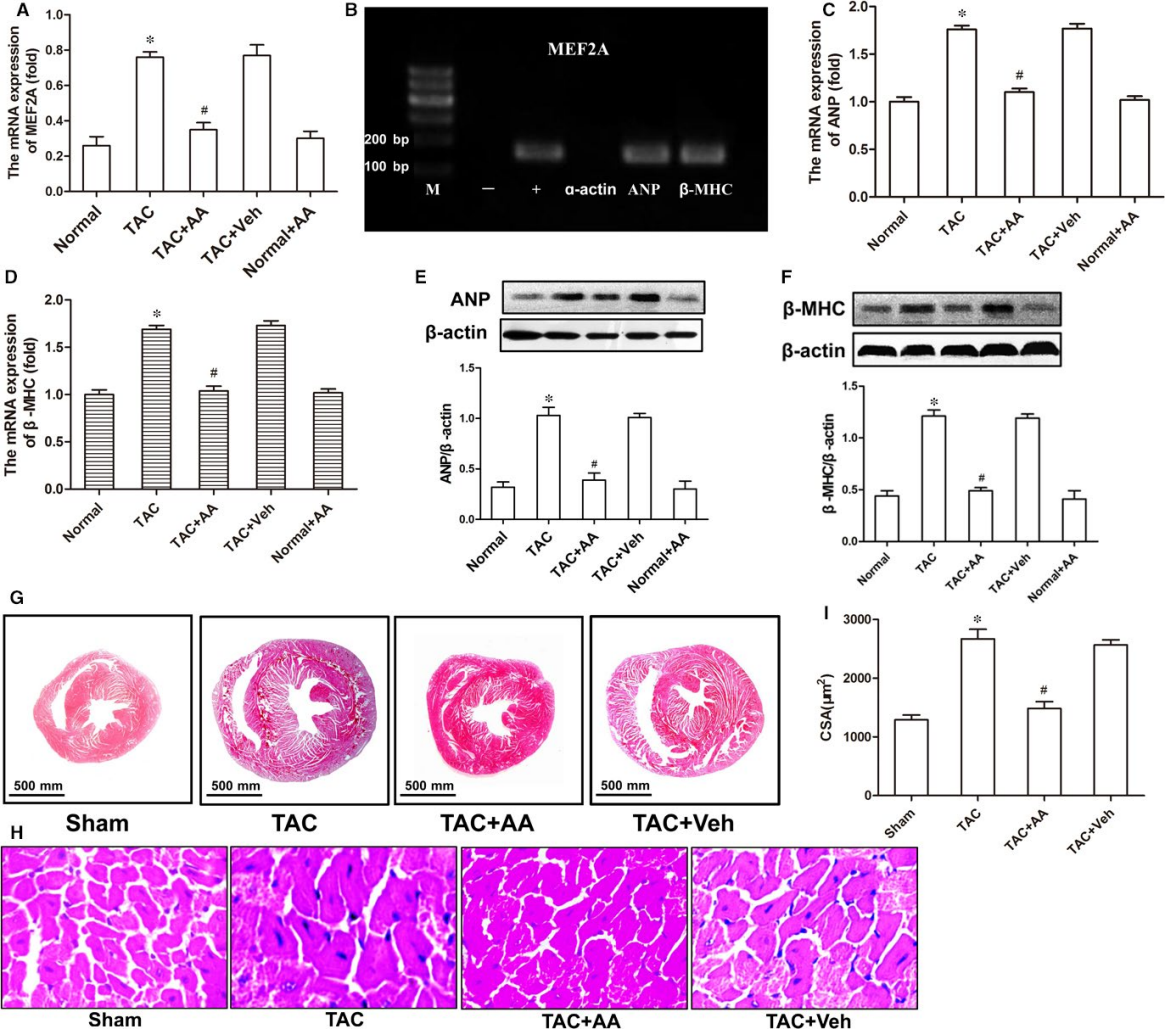
Anacardic acid (AA) attenuates the overexpression of cardiac hypertrophy‐linked genes in transverse aortic constriction (TAC) mice. (A) Q‐PCR analyses were used to determine the mRNA expression of *MEF2A*. (B) ChIP‐PCR assays were used to identify the regulatory relationships among MEF2A, atrial natriuretic peptide (ANP) and myosin heavy chain (β‐MHC), which clearly showed the binding of MEF2A at the promoter region of ANP and β‐MHC, but MEF2A did not bind to the promoter region of α‐actin. (C and D) Q‐PCR analyses of the mRNA levels of ANP and β‐MHC in the mouse heart tissues. (E and F) Western blots showing the expression of ANP and β‐MHC at the translational level in the mouse hearts. (G and H) Haematoxylin and eosin staining of cardiac tissue in the mouse hearts. (I) The cross‐sectional area of cardiomyocytes. *P < 0.05 vs the normal group. ^#^ *P* < 0.05 vs the TAC group (n = 6)

*MEF2A* is a critical transcription factor that is involved in heart development, cardiac hypertrophy and many other cardiovascular diseases. Thus we first tested the mRNA expression of the *MEF2A* gene through Q‐PCR and found an obvious increase in gene expression in TAC mice, while exposure to AA decreased the overexpression of *MEF2A* mRNA in the TAC mouse hearts (Figure 4A). To *MEF2A* on cardiac hypertrophy‐related downstream genes in TAC mice, we assayed the regulatory relationship between MEF2A and downstream cardiac hypertrophy‐linked genes (*ANP* and *β‐MHC*). The binding affinity between MEF2A and the promoters of *ANP, β‐MHC *and* α‐actin* was detected by PCR after ChIP. The ChIP‐PCR results showed that MEF2A could bind to the promoters of *ANP* and *β‐MHC* but not *α‐actin *(Figure 4B). The above results indicate that the heart nuclear transcription factor *MEF2A* is involved in regulating cardiac hypertrophy‐related *ANP* and *β‐MHC *gene expression.

To investigate the effects of AA on cardiac hypertrophy in the hearts of TAC mice, we assayed the biomarkers of myocardial hypertrophy, ANP and β‐MHC, by Q‐PCR and immunoblotting, and the morphology of hearts in the TAC mice was also examined using haematoxylin and eosin staining. Q‐PCR data showed that the mRNA levels of *ANP* and *β‐MHC* in the hearts of TAC mice treated with AA were significantly decreased compared to those of TAC mice treated with Veh (Figure 4C,D). The Western blot results showed that AA could also attenuate the overexpression of ANP and β‐MHC in the same samples (Figure 4E,F). Haematoxylin and eosin staining data showed that AA could significantly reduce the left ventricle and ventricular septum thickness in the hearts of TAC mice (Figure 4G). The cross‐sectional area of cardiomyocytes in the TAC + AA group was apparently diminished compared to that of the TAC group (Figure 4H,I).

### 3.7 AA improves survival rate and cardiac function in the hearts of TAC mice

**Figure 5:**
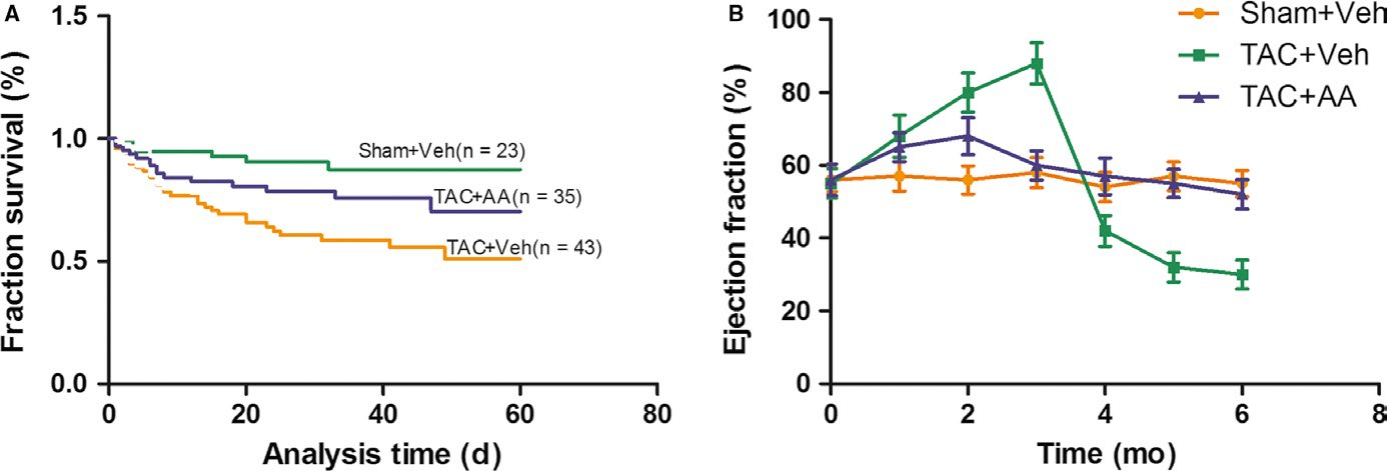
Survival rate and ejection fraction in transverse aortic constriction (TAC) mice. Survival rate in TAC mice treated with anacardic acid (AA) or Veh. (B) Left ventricular ejection fraction in the hearts of TAC mice treated with AA or Veh (n = 6)

For clinical use of the HAT inhibitor AA, it is important to evaluate its long‐term efficacy and tolerability. To explore this issue, we studied mice subjected to TAB or sham operation and treated them with AA (3.75 mg/kg, every 3 days) for 8 weeks, a period roughly corresponding to 6 to 8 years in humans. In this study, exposure to AA was well tolerated throughout the study (8 weeks) and had no effect on survival [Sham + Veh, 95% (n = 23); TAC + Veh, 45% (n = 43); TAC + AA, 73% (n = 35)] (Figure 5A). The current results suggest that AA can suppress hypertrophic growth.

To examine the effect of AA on the normal functioning of hearts, we performed echocardiography of the mice. Here, we observed modest declines in left ventricular end diastolic volume (LVEDV), left ventricular end systolic volume (LVESV), left ventricular end diastolic dimension (LVEDD) and left ventricular end systolic dimension (LVESD) in TAC + Veh mice, which is consistent with pressure overload‐mediated pathological reformation. In mice treated with TAC + AA, there was a sharp and significant decline in LVEDV, LVESV, LVEDD and LVESD expression compared to that of TAC + Veh mice (*P < *0.05), despite the presence of blunted but compensational hypertrophic responses. Meanwhile, the interventricular septum (IVS) and left ventricular posterior wall (LVAWT) were decreased significantly in TAC + AA mice compared with those of TAC + Veh mice (Table 2). Additionally, LVEF was observed in the mouse hearts, and the data showed that LVEF was increased significantly in TAC mice as time increased, but after 4 months of operation, LVEF was apparently decreased in TAC mice, perhaps due to heart failure. Importantly, LVEF in the TAC mice treated with AA had no apparent change compared with that of the sham group, and heart failure was not observed in the TAC mice treated with AA (Figure 5B).

**Table 2:** Cardiac function measurements with echocardiography (n = 6)

	Normal	TAC	TAC + Veh	TAC + AA	*F* value	*P* value
Parameters						
Body weight (g)	38.57 ± 2.99	39.13 ± 4.32	38.52 ± 2.76	37.36 ± 3.46	3.51	0.17
Heart rate (bpm)	468 ± 5	476 ± 8	464 ± 5	443 ± 4	2.57	0.26
LV end diastole						
LVAWT (mm)	1.02 ± 0.04	1.62 ± 0.05	1.69 ± 0.04	1.23 ± 0.05**	5.76	0.04
IVS (mm)	0.71 ± 0.03	1.02 ± 0.03*	1.05 ± 0.02	0.76 ± 0.02**	6.89	0.03
LVEDD (mm)	3.54 ± 0.05	2.58 ± 0.05*	2.67 ± 0.03	2.91 ± 0.04**	5.98	0.04
LV volume (μL)	47.88 ± 0.40	35.46 ± 0.51*	48.02 ± 0.54	38.66 ± 0.43**	6.92	0.03
LV end systole						
LVAWT (mm)	1.32 ± 0.06	1.76 ± 0.04*	1.80 ± 0.02	1.42 ± 0.03**	5.39	0.04
IVS (mm)	0.99 ± 0.03	1.03 ± 0.01	1.01 ± 0.05	1.00 ± 0.02	2.15	0.28
LVESD (mm)	2.29 ± 0.02	1.67 ± 0.03*	1.58 ± 0.02	2.09 ± 0.02**	5.75	0.04
LV volume (μL)	22.54 ± 0.34	12.51 ± 0.28*	11.18 ± 0.22	19.28 ± 0.32**	5.66	0.04

AA, anacardic acid; IVS, interventricular septum; LVAWT, left ventricular anterior wall thickness; LVEDD, left ventricular end diastolic dimension; LVESD, left ventricular end systolic dimension; TAC, thoraic aorta coarctation.

* *P* < 0.05 TAC vs normal group.

** *P* < 0.05 TAC + AA vs TAC + Veh.

## 4 DISCUSSION

In this study, we demonstrated that pressure overload‐mediated cardiac hypertrophy in TAC mice, in which histone acetylation played a significant role in myocardial hypertrophy. Additionally, we demonstrated that AA, a Chinese herbal extract, could attenuate cardiac hypertrophy by repressing HAT function, subsequently decreasing histone acetylation.

In fact, haemodynamic stress has been suggested to cause cardiac hypertrophy, resulting in cardiac malfunctioning and abrupt failure.^11^, ^12^, ^13^ This process corresponds to the expression of genes induced by epigenetic regulation involved in postmitotic myocytes, leading to cardiac hypertrophy. This epigenetic alteration in response to extrinsic stresses promoted alterations in the translation of sarcomeric protein, relaxation of diastolic turns, metabolism, modulation of calcium influx and formation of collagen, which are solely controlled by various transcriptional regulators of the stress‐response muscle enhancer factor‐2 (MEF2) family.^14^, ^15^ To date, many distinct HATs have been reported in humans. Five relatively important HAT isoforms have been reported in mammalian cardiac cells—p300, CREB‐binding protein, steroid receptor coactivator‐1, PCAF and general control nonderepressible‐5 (GCN5).^16^ In our study, we clearly demonstrated that p300‐HAT and PCAF‐HAT in cardiomyocytes are vital regulatory factors. Acetylated H3K9 on the promoter of *MEF2A,* a cardiac hypertrophy‐related transcriptional regulator, caused overexpression of cardiac hypertrophy‐related genes, eventually inducing the development of cardiac hypertrophy.

The HAT activity could be inhibited pharmacologically to increase or decrease related gene expression accordingly.^17^, ^18^, ^19^ HAT inhibitors such as AA, curcumin and ANP32A have attracted attention in recent years by cardiologists as they can repress histone acetylation.^20^, ^21^, ^22^ HAT and HDAC are two key histone modulators known to play crucial roles in survival, growth, proliferation and cellular differentiation that participate in cardiac myocyte growth and help produce stress responses.^23^, ^24^ A recent study confirmed that HAT p300 is required for cardiac myocyte gene expression.^6^ Our results showed that the imbalance of histone acetylation (H3K9ac) induced by p300 and PCAF (isoforms of HATs) could be involved in promoting myocardial hypertrophy in the hearts of TAC mice. We also showed that *MEF2A *mRNA expression was significantly increased in the hypertrophic hearts of TAC mice. These results suggest that *MEF2A* is a vital transcription factor contributing to myocardial hypertrophy by pressure overload. However, AA administration could inhibit *MEF2A* transcription by decreasing H3K9ac hyperacetylation through repression of PCAF and p300 at the *MEF2A* promoters*.* Interestingly, AA also normalized the overexpression of myocardial hypertrophy biomarker genes, for example, ANP and β‐MHC, in the cardiac tissue of TAC mice. Our data suggest that H3K9 hyperacetylation is mediated by PCAF‐HAT and p300‐HAT and plays a significant role in pressure overload‐induced cardiac hypertrophy. The findings are consistent with previous reports that the imbalance of histone H3K9ac modification induced by p300 and PCAF is involved to involve in pathological cardiac hypertrophy.^25^, ^26^

In our previous studies, the imbalance in acetylation of histones facilitated by HAT (PCAF, p300) was related to cardiac hypertrophy‐mediated phenylephrine.^27^ This study demonstrated that hyperacetylation of H3K9ac induced by HAT (p300 and PCAF) is involved in the development and progression of cardiac hypertrophy caused by TAC. The findings of this study suggested that HAT activation was involved in the final and joint cellular pathways driven via varied hypertrophic agonists. Inhibition of gene activation and expression at this step may be a therapeutic strategy for preventing or ameliorating cardiac hypertrophy, which subsequently leads to heart failure. AA has been reported to inhibit HAT activity.^28^ Our experiments demonstrated that AA could also inhibit HAT to attenuate myocardial hypertrophy, especially that of the left ventricular wall and ventricular septum, and echocardiography data further demonstrated that AA could improve cardiac function. Meanwhile, long‐term exposure to AA improved the survival rate along with cardiac function in TAC mice, and no toxic or side effects were observed in TAC mice treated with AA. However, the development of a new HAT inhibitor may result in increased selectivity and decreased toxic effects. Additional pre‐clinical studies are needed to confirm that this novel HAT inhibitor can be used for the prevention or reversal of cardiac hypertrophy as well as heart failure. Previously, we reported^27^ that some related indices in blood samples and no any abnormalities in AA‐treated mice were observed, which suggested and confirmed that AA could be a candidate drug for further clinical experiments. However, large studies and more data are needed prior to the application of AA in clinical trials.

AA attenuates cardiac hypertrophy mediated by pressure overload by inhibiting HAT activity, which may contribute to reducing and treating hypertrophy through cardiomyopathy. However, the upstream signalling pathway of AA‐attenuated cardiac hypertrophy in TAC mice is still unknown. In addition, in the process of constructing the cardiac hypertrophy model by TAB in mice, the mortality rate is approximately 40%, and the main causes of death include infection, surgical trauma, heart failure and shocks. We used only the surviving mice for the experiments. Nevertheless, our current results provide strong evidence suggesting that inhibition of HAT is a potential therapeutic strategy and show promise for treating overload‐induced heart dysfunction.

## CONFLICT OF INTEREST

The authors have no conflicts of interest to declare.
